# Medical News and Miscellany

**Published:** 1890-03

**Authors:** 


### Medical News and Miscellany

THE KENTUCKY SCHOOL OF MEDICINE (Spring courses) is booming. The class numbers 300, and "the cry is still they come." Write to Prof. Wathen, Dean, Louisville, Ky., for catalogue.

GAILLARD'S MEDICAL JOURNAL for March is an exceptionally fine number of that always good journal. The editorial on the Heredity of Greatness is a superior piece of composition, full of original thought and feeling.

FOR SALE OR RENT.—A well stocked drug store and practice in a town of about a thousand inhabitants. Good reason for selling. For particulars address A. A. Wadgymar, Carrizo Springs, Dimmit county, Texas.

PERSONALS, REMOVALS, ETC.—We learn that Dr. W. T. Baird has removed from El Paso to Dallas and formed co-partnership with Dr. O. L. Williams, at Oak Cliff, Dallas, Dr. Williams having recently removed from Chappel Hill to Dallas.

Dr. H. S. Robertson, who left Lockhart some two years ago, and settled in Prescott, Arkansas, has returned to Lockhart.

Dr. W. J. Matthews, of this city, has taken the degree of M. D. at the University of Tennessee, where he attended a course of lectures the past season.

Dr. F. M. D. Hill has removed from Cat Spring to Prairie Plains.

REMOVALS.—Dr. A. J. Cammack has removed from Bosqueville to Waco, and Dr. R. E. Thompson has located at Bosqueville.

Dr. J. L. Cunningham, one of the foremost sanitarians in Texas, having accomplished the hygienic regeneration of Dallas, has removed to Fort Worth. He has begun the siege, and is doing good missionary work; but we fear he will find the Fort an Augean stable. Success to him, however.

Dr. P. K. Wortham has removed from Meridian to Waco.

---

FROM FAR-OFF WASHINGTON.—Dr. R. L. Thompson, late managing editor of the St. Louis Weekly Medical Review, and Secretary of the Mississippi Valley Medical Association, has, we learn, removed to Spokane Falls, Washington, and will join our quondam Texas confrere, Dr. Wm. Caston, in the special practice, "Eye, Ear, Nose and Throat." Dr. Caston, it will be remembered, lost his outfit in the late disastrous fire at Spokane; but he writes us he is O. K. once more and ready for any emergency. The Doctor was largely instrumental in procuring the passage of an act to regulate the practice in Washington lately, and we have had some intimation that he and Dr. Thompson will shortly establish a medical journal in Washington. Verily, Texas is getting badly left in the one thing needful,—medical legislation.

---

PERSONALS—Dr. B. D. Smith, a graduate of the Southern Medical College, Atlanta, has located at St. Elmo, this county.

Dr. T. J. Wagley, of Cleburn, a prominent member of the State Medical Association and a staunch friend of the JOURNAL, sailed for Berlin, via London, on the 26 ultimo. He is accompanied by his accomplished wife, and they will make an extended tour of Europe, spending a fortnight in London. Dr. Wagley writes: "I could not think of being deprived of my favorite journal during my absence from Texas, so please have it sent to me at Berlin, care of Mendelsohn & Co., Bankers, till further notice." The doctor will attend the celebrated clinics and hospitals of Germany and Prussia, and when he comes back we expect him to speak nothing but German. Good luck to him; we shall miss him at Fort Worth.

Dr. R. B. White, of Ennis, a member of the State Medical Association, died February 10 ultimo. The Association is losing an unusually large number of members lately.

Dr. R. F. Minnock, of Bosqueville, is at the New York Polyclinic.

Dr. A. C. Walker, of Rockdale, performed laparotomy for gunshot wound of the bladder in a man of twenty-one. Sutured the bladder (omitting the mucous membrane) and saved his patient. We believe this is the first case on record of successful suturing of the bladder for gun-shot wou.ıd. The case will appear in full in our next issue.

### SOME RECENT ADDITIONS TO THE MATERIA MEDICA.

Among the new drugs which Messrs. Parke, Davis & Co. announce they can supply are the following:

### COCILLANA.

Guare—Species indetermined. Synonym—Sycocarpus rusbyi, Britton. Part employed—The bark. Natural order—Anacardiacea. Habitat—Bolivia. Properties—Expectorant, tonic, laxative.

This new remedy possesses a sphere of influence on the respiratory organs somewhat similar to ipecac, but said to be "superior in certain diseases of the air passages in which the latter is often used." Dr. D. D. Stewart in Medical News, August 24, 1889.

Besides its excellence as an expectorant, clinical experience has also established the fact that it exerts a tonic influence upon the appetite, and that it reduces the night sweats of chronic bronchitis and phthisis. Cocillana also gives promise of usefulness as a laxative. Dose—Ten to thirty minims 9. 6 to 2 C. C.

### ESCHSCHOLTZIA.

Eschscholtzia California, cham. Synonym—California poppy.

Part employed—The whole plant. Natural order—Papaveraceæ. Habitat—California.

Properties—"An excellent soporific and analgesic, and above all harmless."

Recent analysis claims to have discovered the presence of a minute quantity of morphine in the plant. The quantity contained, however, is not sufficient to account for all the therapeutic effects, and further chemical investigation promises to isolate another active principle which may better explain its action.

The drug is a very useful anodyne in certain cases. The inconveniences attributed to the use of opium, such as stomach disturbance, constipation, etc., have not in any case been observed in its use. It may with advantage replace opium preparations for children.

Fluid extract of the plant. Dose, 15 to 30 minims [1 to 2 C.C.]

### JATROPHA.

Jatropha macrorhiza; benth. Synonyms—Jicama, jicomia, span. Part employed—Euphobi aceæ, Habitat—Northern Mexico and southern states adjoining.

Properties—Alterative and cholagogue ; in larger does hydrocathartic and sometimes emetic. Jatropha macrorhiza, a household remedy of the Mexicans, has been recently recommended for use in this country by Dr. A. H. Noon, on account of its comparative tastelessness—the slight taste the drug possesses being compared to that of the sweet potato. It has been suggested as an addition to non-cathartic, but otherwise astringent mixtures, its use could not be otherwise than valuable. Clinical experience will doutless develop other and more specific indications for its employment.

Fluid extract of the root. Dose, 1-2 to 2 Fluiddrachms [2 to 8 C. C.]

### HYDRASTININE

A new derivative of hydrastine. A possible substitute for ergot.

This substance, an oxidation product of hydrastine, white alkaloid of golden seal, has recently been prepared by them in order to afford opportunities for physiological investigation in European laboratories, prominent among which are those of the Universities of Dorpat and Berlin.

It can be obtained from hydrastine by the action of various oxidizing agents, and though the original methods were attended with considerable waste, improvements in this respect are constantly being made. So far the most troublesome element is encountered in its purification and crystalization. The reaction taking place in its production may be illustrated thus :

| HYDRASTINE. | HYDRASTININE. | OPIANIC ACID |
|---|---|---|
| 21 H 21 NO. 6. | C 11 H 11 NO 2. | C 10 H 10 O 5. |

The alkaloid, or base, being sparingly soluble and moreover rather prone to decomposition when in solution. We have given preference to the hydrochlorate as possessing the desirable elements of stability and solubility in aqueous fluids.

Recent advices from the highest European authorities represent it to be of immeasurable service in controlling uterine hemorrhages, far surpassing ergot in efficiency, certainty of action and safety.

---

The New York Pharmacal Company of Yonkers, have lately supplied the profession with some valuable literature testifying to the value of that staunch stand-by, Lactopeptine. If this company would just substitute the picture of a handsome dude—such as we see in some drug stores—for that fat pig, which evidently is not even a distant relative of the pig of history—Jack Spratt's pig—we would like it, but perhaps it would not so well illustrate the digestive powers of Lactopeptine. Now Jack Spratt's pig—so the history says—"was not very fat—nor yet very lean." If he had been made to stand on his hind feet and pulverize Lactopeptine in a mortar, as long as this pig has done, no doubt he would have been as round and saucy as he,—but, as warm weather will be here by and by, it is as well to bear in mind that in cholera infantum, Lactopeptine is a tower of strength. Do you remember Dr. Cunningham's letter on cholera infantum? Forewarned is forearmed.

